# Supplementary material for: The IL-33-ILC2 pathway protects from amebic colitis
Source: Mucosal Immunol. 2021 Aug 16;15(1):165–75. doi: 10.1038/s41385-021-00442-2 (PMC8732277; doi:10.1038/s41385-021-00442-2)
Supplement: Supplementary file 1 — Supplementary Figures 1–7 [file 41385_2021_442_MOESM1_ESM.pdf]

Supplementary figure 1

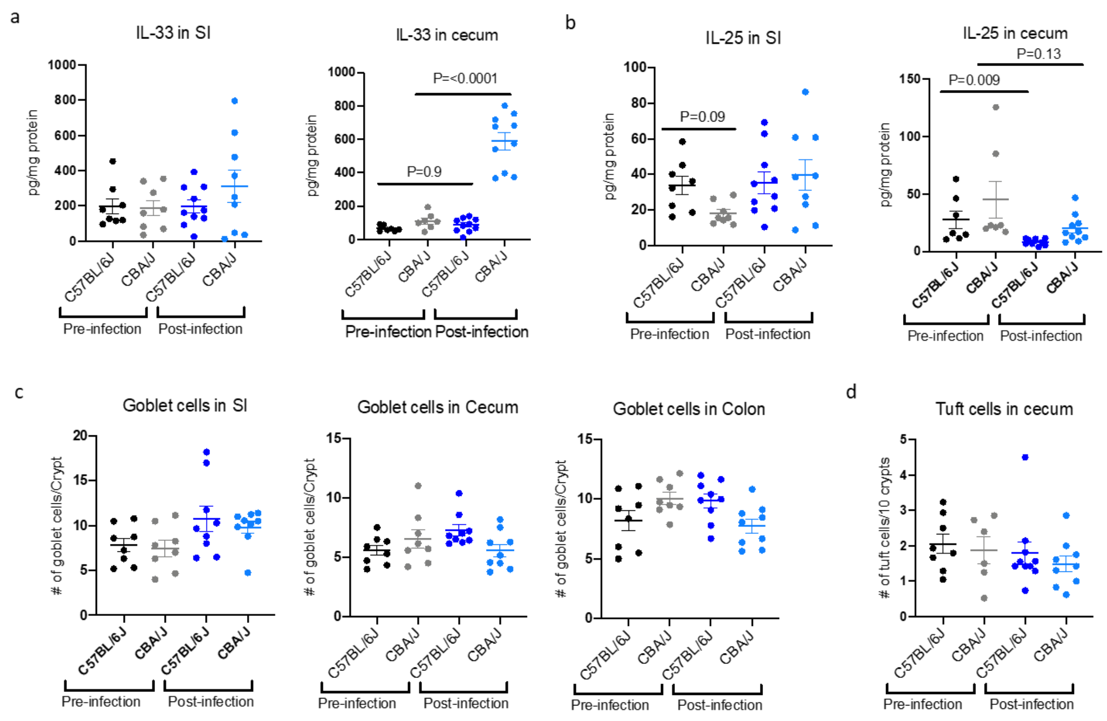

Supplementary figure 1: **There are no significant differences in IL-33 and IL-25 protein between C57BL/6 and CBA/J mice at baseline.** C57BL/6J and CBA/J strain of mice were euthanized before the amebic challenge and on day 3 of the amebic challenge. Tissue sections from the small intestine (SI) and cecum were collected, and tissue lysates were prepared. The concentration of IL-33 and IL-25 were measured from tissue lysate using a multiplex Luminex assay. Tissue sections from SI, cecum, and colon were also fixed in Bouin's solution, followed by staining for goblet cells. Tissue sections from the cecum were also stained for tuft cells. **a** IL-33 protein in SI and cecal tissue lysate, n=7-10 mice per group. **b** IL-25 protein in SI and cecal tissue lysate, n=7-10 mice per group. **c** PAS-stained goblet cells in SI, cecum, and colon, n=8-10 mice per group. **d** Number of tuft cells in the cecum determined by DCAMKL1 staining, n=6-10 mice per group**.** Statistical significance was determined by one-way ANOVA. Error bars indicate SEM.

Supplementary figure 2

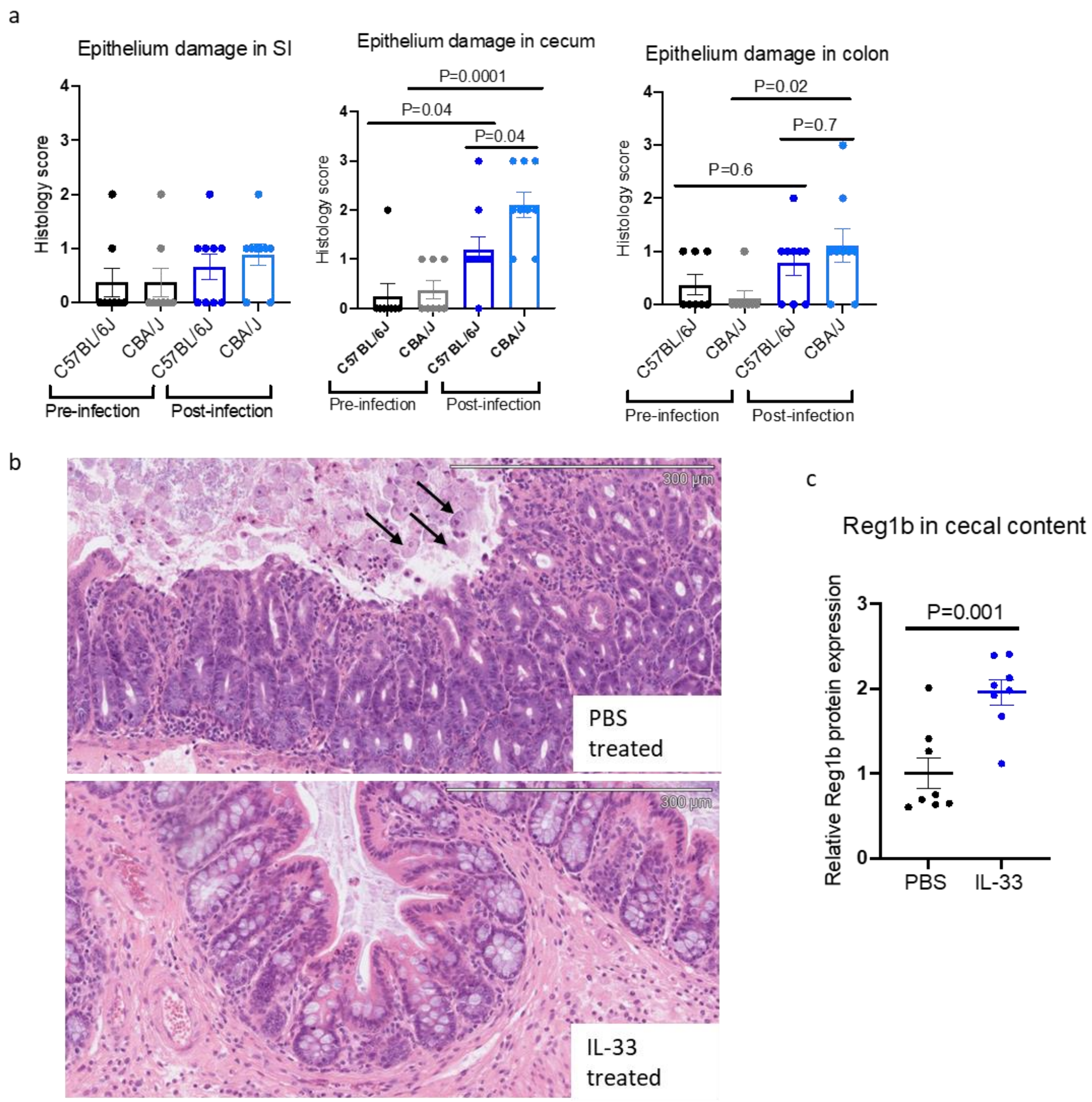

Supplementary figure 2: ***E. histolytica*** **infection induces epithelial tissue damage in the cecum and colon but not in SI. a** C57BL/6J and CBA/J strain of mice were euthanized prior to and on day 3 of the amebic challenge. Tissue sections from the cecum, colon, and SI were fixed in Bouin,s solution, followed by H&E staining. Epithelial damage was scored in the range from 0-3, n=7-10, per group. **b-c** CBA/J mice were injected intraperitoneally with 0.75 µg of IL-33 or PBS each day for eight days. On day 4, mice were challenged with *E. histolytica* trophozoites. Cecal tissue and cecal content were harvested on day 9 (day

5 of *E. histolytica* challenge). **b** H&E stained section shows epithelial damage in PBS treated mice but not in IL-33 treated mice. Arrows show amebic trophozoites are identified near damaged epithelium. **c** Reg1b protein expression measured by ELISA from cecal content. Statistical significance was determined by one-way ANOVA and unpaired t test. Error bars indicate SEM.

Supplementary figure 3

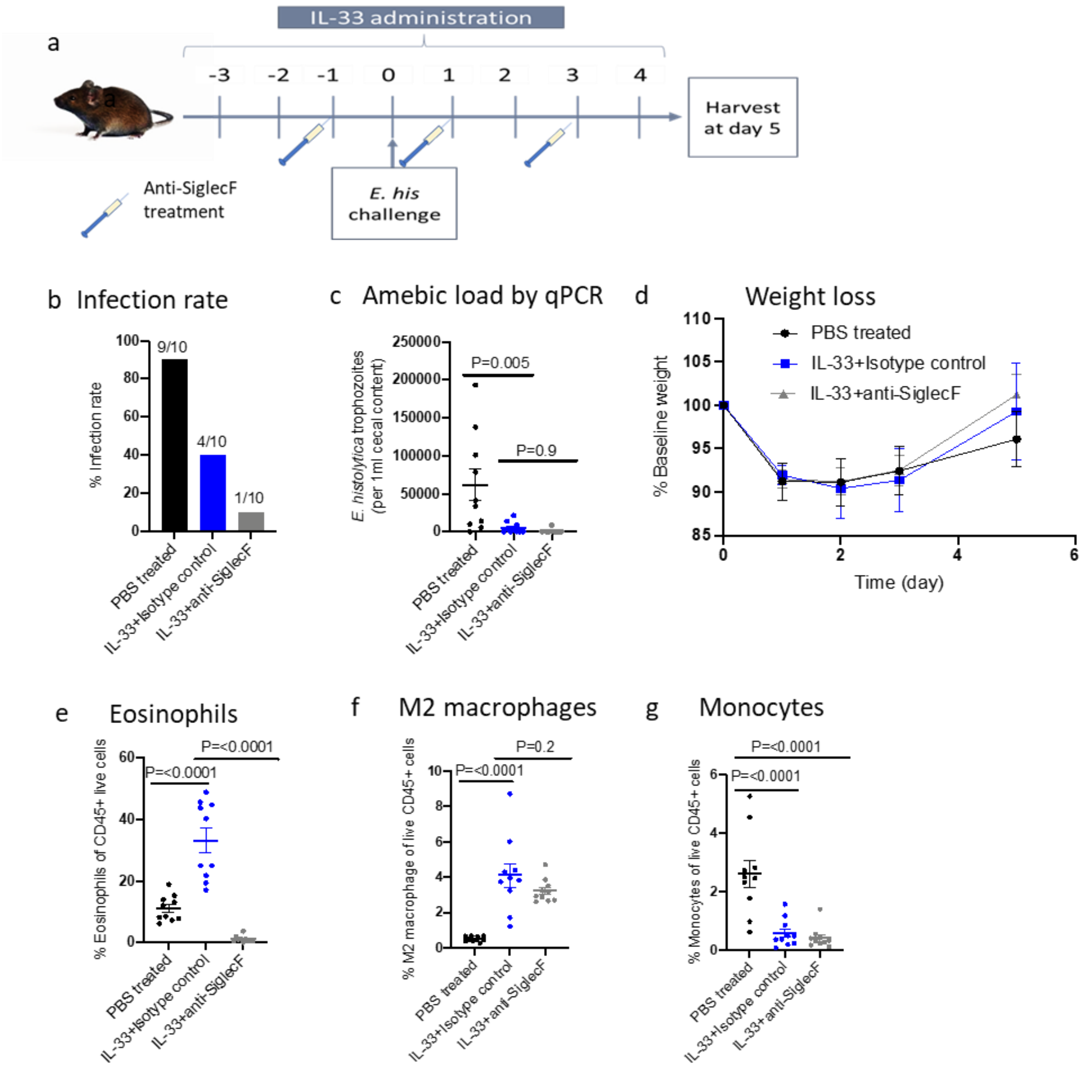

Supplementary figure 3: IL-33 mediated protection from amebic colitis is not mediated by eosinophils. **a-g** CBA/J mice were treated with 0.75 µg of IL-33 or PBS for 8 days. IL-33 treated groups were administered 3 doses of anti-SiglecF antibody or isotype control. **a** Experimental outline. **b** Infection rate

by amebic culture. **c** Amebic load by qPCR**. d** Weight loss. **e-f** Percentage of eosinophils, CD206+ macrophages, and monocytes in cecal tissue. **b-g** n=10, per group. Statistical significance was determined by one-way ANOVA. Error bars indicate SEM.

Supplementary figure 4

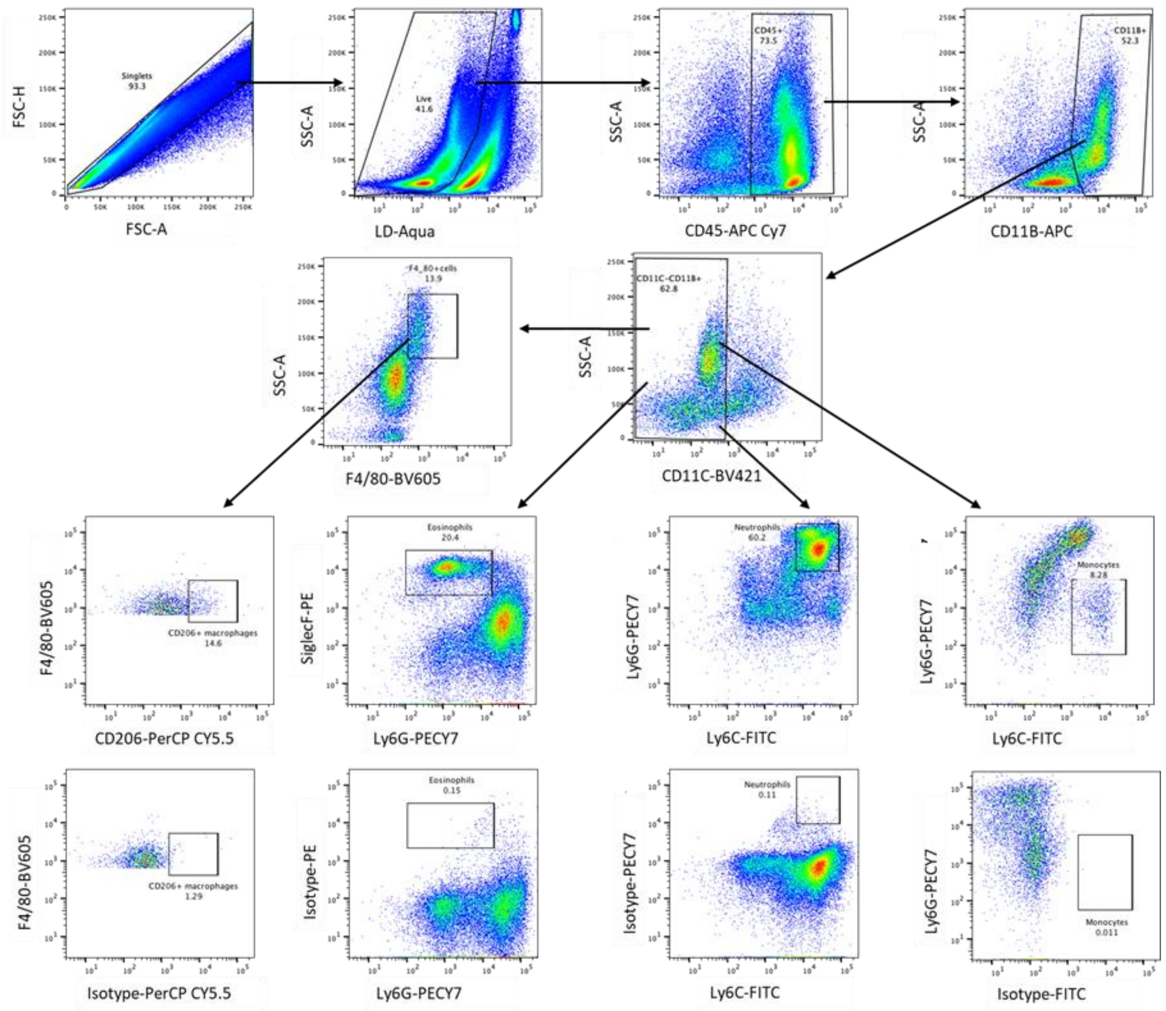

Supplementary figure 4: Flow cytometry gating strategy to determine eosinophils, neutrophils, monocytes, and M2 macrophages in the cecum. Flow cytometry gating was determined based on FMO (fluorescent minus one) control. Cells were identified using following strategy- eosinophils:

CD45+CD11B+CD11C-SiglecF+, neutrophils: CD45+CD11B+CD11C-Ly6G^{hi}, monocytes: CD45+CD11B+CD11C-Ly6C^{hi}, m2 macrophages: CD45+CD11B+CD11C-F4/80+CD206+.

Supplementary figure 5

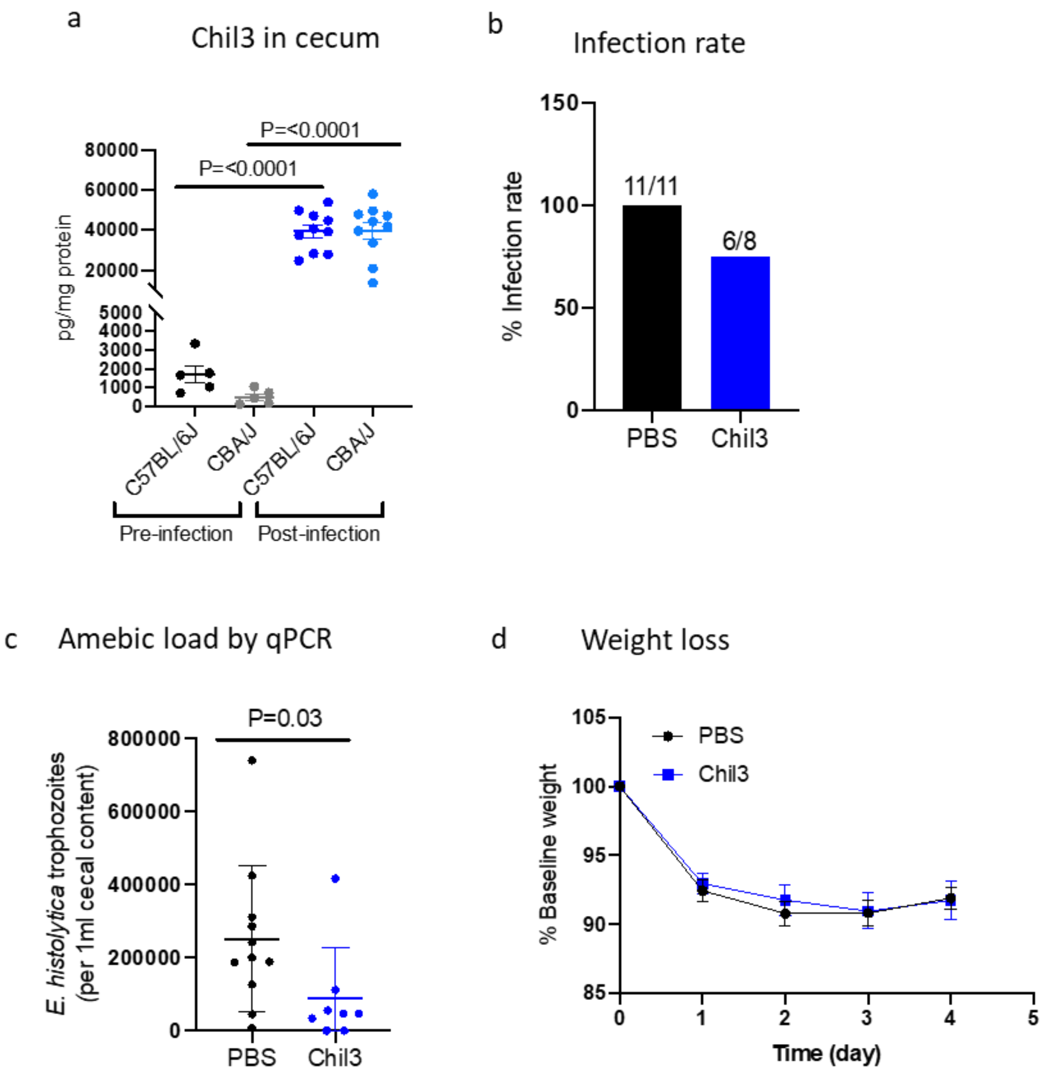

Supplementary figure 5: Treatment with Chil3 protects from amebic infection. a C57BL/6J and CBA/J strain of mice were harvested before the amebic challenge and on day 3 of the amebic challenge. Chil3 protein was measured by ELISA from cecal tissue lysate. **b-d** CBA/J mice were administered 6 doses (on day -2, -1, 0, +1, +2 +3) of recombinant Chil3 (5ug/dose) via intraperitoneal injection. On day 5 of the

amebic challenge, mice were harvested to collect cecal tissue and cecal content. **b** Infection rate by amebic culture. **c** *E. histolytica* DNA, measured by qPCR. **d** Weight loss. **a** N=5-10 mice per group. **b-d** N=8-11 mice per group. Statistical significance was determined by one-way ANOVA and unpaired t-test. Error bars indicate SEM.

Supplementary figure 6

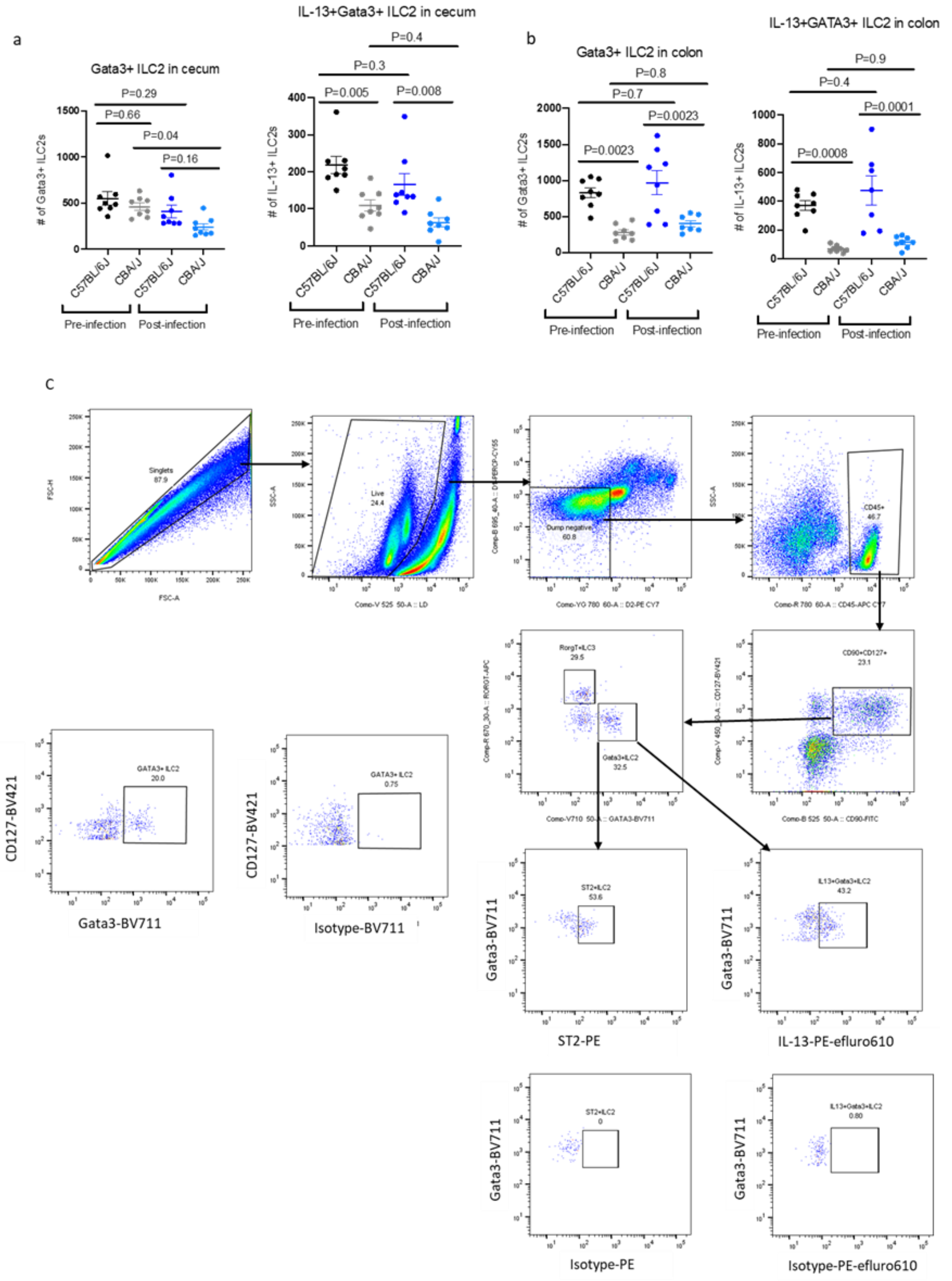

Supplementary figure 6: C57BL/6J mice have a significantly higher number of IL-13 producing ILC2s compared to CBA/J mice at baseline and after amebic infection. a-b C57BL/6J and CBA/J strain of mice were euthanized before and on day 3 of the amebic challenge. Single cells were isolated from cecal tissue and colonic tissue followed by flow-staining with antibodies to detect IL13+Gata3+ILC2s. **a** Total number of Gata3+ILC2s, and IL13+Gata3+ILC2s in the cecum, n=8 mice per group. **b** Total number of Gata3+ILC2s and IL13+Gata3+ILC2s in the colon, n=7-8 mice per group. **c** Flow cytometry gating strategy to determine ILC2s. Lineage cells (CD19+, CD3+, CD5+, CD11c+, CD11b+, FcεR+) were gated out from live cells. The following gating strategy was then used to determine ILC2s: Lin^- CD45+ CD90+ CD127+ GATA3+. The GATA3+ cells were then gated for ST2 and IL-13**.** Statistical significance was determined by one-way ANOVA. Error bars indicate SEM.

Supplementary figure 7

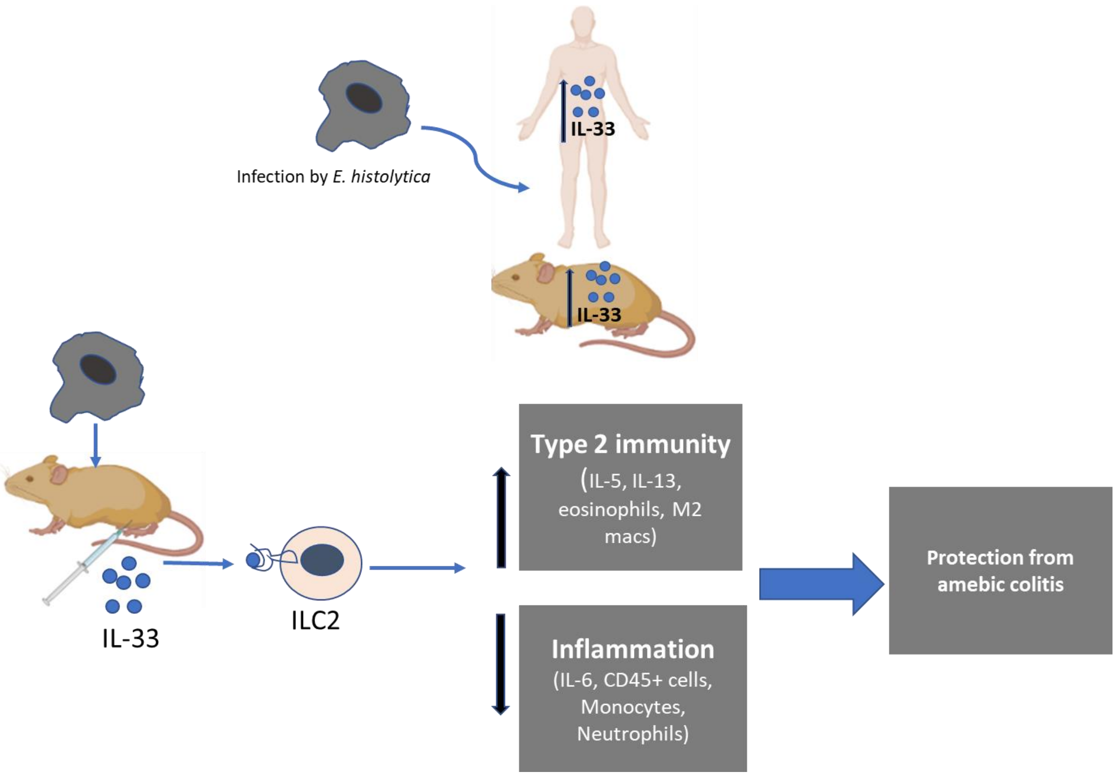

Supplementary figure 7: Graphical summary: The IL-33-ILC2 mediated protection from amebic colitis. *E. histolytica* infection upregulates IL-33 expression in human and mouse colon (upper figure). Exogenous IL-33 acts on ILC2s to induce a type 2 immunity and dampen overall inflammation resulting in protection from amebic colitis (bottom figure). The figures were generated using the BioRender.com and the Microsoft PowerPoint.
